# Supplementary material for: Corrected and Republished from: “A Novel, Multiple-Antigen Pneumococcal Vaccine Protects against Lethal Streptococcus pneumoniae Challenge”
Source: Infect Immun. 2022 Jan 25;90(1):e00846-18a. doi: 10.1128/IAI.00846-18a (PMC9199499; doi:10.1128/IAI.00846-18a)
Supplement: Supplemental file 1 — Supplemental material. Download IAI.00846-18-s0001.pdf, PDF file, 0.2 MB [file iai.00846-18-s0001.pdf]

## Supplementary Table 1

**All proteins that iTRAQ analyses show have increased expression in the MAV TIGR4.1 compared to the TIGR4 HKL preparation**

| SP # | Protein | Fold change |
|---|---|---|
| SP_0082 | Cell wall surface anchor family protein (PavB) | 5.33 |
| SP_0148 | ABC transporter, substrate-binding protein | 3.10 |
| SP_0149 | Lipoprotein | 2.52 |
| SP_0285 | Alcohol dehydrogenase, zinc-containing | 2.90 |
| SP_0338 | Putative ATP-dependent Clp protease, ATP-binding | 2.67 |
| SP_0409 | Putative uncharacterized protein | 2.96 |
| SP_0447 | Ketol-acid reductoisomerase (IlvC) | 2.66 |
| SP_0466 | Putative sortase | 3.42 |
| SP_0516 | Protein GrpE (GrpE) | 3.03 |
| SP_0517 | Chaperone protein (DnaK) | 4.33 |
| SP_0629 | Putative uncharacterized protein | 3.94 |
| SP_0641 | Serine protease, subtilase family | 3.12 |
| SP_1027 | Putative uncharacterized protein | 3.12 |
| SP_1190 | Tagatose 1,6-diphosphate aldolase (LacD) | 4.74 |
| SP_1192 | Galactose-6-phosphate isomerase subunit (LacB) | 3.13 |
| SP_1415 | Glucosamine-6-phosphate deaminase (NagB) | 2.62 |
| SP_1572 | Non-heme iron-containing ferritin | 2.6 |
| SP_1650 | Manganese ABC transporter lipoprotein (PsaA) | 4.03 |
| SP_1676 | Putative N-acetylneuraminate lyase | 3.06 |
| SP_1775 | Conserved domain protein | 2.79 |
| SP_1776 | Thioredoxin (Trx) | 2.96 |
| SP_1906 | 60 kDa chaperonin (GroL) | 2.95 |

| Supplementary Table 1 continued | | |
|---|---|---|
| **SP #** | **Protein** | **Fold change** |
| SP_1907 | 10 kDa chaperonin (GroS) | 3.56 |
| SP_2055 | Alcohol dehydrogenase, zinc-containing | 3.46 |
| SP_2056 | N-acetylglucosamine-6-phosphate deacetylase | 3.24 |
| SP_2093 | Putative uncharacterized protein | 2.78 |
| SP_2108 | Maltose/maltodextrin-binding protein (MalX) | 3.19 |
| SP_2239 | Serine protease | 3.29 |
| SP_1069 | Putative uncharacterized protein | 2.51 |
| SP_2126 | Dihydroxy-acid dehydratase (IlvD) | 2.48 |
| SP_0519 | Chaperone protein (DnaJ) | 2.37 |
| SP_0845 | Lipoprotein | 2.37 |
| SP_1500 | Amino acid ABC transporter, (AatB) | 2.37 |
| SP_1527 | Oligopeptide-binding protein (AliB) | 2.33 |
| SP_0771 | Peptidyl-prolyl cis-trans isomerase, cyclophilin-type | 2.31 |
| SP_1683 | Sugar ABC transporter, sugar-binding protein | 2.3 |
| SP_0899 | Putative uncharacterized protein | 2.26 |
| SP_2070 | Glucose-6-phosphate isomerase (Pgi) | 2.24 |
| SP_1032 | Iron-compound ABC transporter | 2.22 |
| SP_0736 | Mannose-6-phosphate isomerase (ManA) | 2.22 |
| SP_0520 | Putative uncharacterized protein | 2.21 |
| SP_0605 | Fructose-bisphosphate aldolase (Fba) | 2.20 |
| SP_2197 | Putative ABC transporter, substrate-binding protein | 2.17 |
| SP_0236 | DNA-directed RNA polymerase subunit alpha (RpoA) | 2.17 |
| SP_0372 | Cell cycle protein GpsB (GpsB) | 2.16 |
| SP_0673 | Putative uncharacterized protein | 2.15 |
| SP_1465 | Putative uncharacterized protein | 2.12 |

| Supplementary Table 1 continued | | |
|---|---|---|
| **SP #** | **Protein** | **Fold change** |
| SP_1996 | Universal stress protein | 2.11 |
| SP_0374 | Putative uncharacterized protein | 2.11 |
| SP_1177 | Phosphocarrier protein HPr (PtsH) | 2.10 |
| SP_1541 | 30S ribosomal protein S6 (RpsF) | 2.09 |
| SP_0499 | Phosphoglycerate kinase (Pgk) | 2.06 |
| SP_1799 | Sugar-binding transcriptional regulator, LacI family | 2.04 |
| SP_0784 | Glutathione reductase (Gor) | 2.03 |
| SP_0231 | Adenylate kinase (Adk) | 2.03 |
| SP_1372 | UPF0342 protein SP_1372 | 2.02 |
| SP_2210 | Cysteine synthase (CysM) | 2.01 |

Only proteins with an increased fold change of 2 and above are shown

## Supplementary Table 2

**All proteins that iTRAQ analyses show have decreased expression in the MAV TIGR4.1 compared to the TIGR4 HKL preparation**

| SP # | Protein | Fold change |
|---|---|---|
| SP_0019 | Adenylosuccinate synthetase (PurA) | -4.00 |
| SP_0032 | DNA polymerase I (PolA) | -2.58 |
| SP_0035 | Aromatic amino acid aminotransferase (AraT) | -2.10 |
| SP_0071 | Zinc metalloprotease ZmpC (ZmpC) | -2.51 |
| SP_0085 | 30S ribosomal protein S4 (RpsD) | -2.56 |
| SP_0095 | UPF0176 protein | -3.09 |
| SP_0103 | Putative capsular polysaccharide biosynthesis | -5.17 |
| SP_0117 | Pneumococcal surface protein A (PspA) | -2.64 |
| SP_0121 | Metallo-beta-lactamase superfamily protein | -2.64 |
| SP_0151 | Methionine import ATP-binding protein MetN | -2.95 |
| SP_0176 | Riboflavin biosynthesis protein RibBA | -3.77 |
| SP_0186 | UvrABC system protein A (UvrA) | -2.80 |
| SP_0202 | Anaerobic ribonucleoside-triphosphate reductase (NrdD) | -2.59 |
| SP_0208 | 30S ribosomal protein S10 | -4.15 |
| SP_0211 | 50S ribosomal protein L23 (RplW) | -2.31 |
| SP_0212 | 50S ribosomal protein L2 (RplB) | -2.90 |
| SP_0215 | 30S ribosomal protein S3 | -4.99 |
| SP_0216 | 50S ribosomal protein L16 | -6.35 |
| SP_0217 | 50S ribosomal protein L29 (RpmC) | -23.94 |
| SP_0219 | 50S ribosomal protein L14 (RplN) | -2.67 |
| SP_0220 | 50S ribosomal protein L24 | -3.69 |

| Supplementary Table 2 continued | | |
|---|---|---|
| SP # | Protein | Fold change |
| SP_0221 | 50S ribosomal protein L5 | -2.08 |
| SP_0222 | 30S ribosomal protein S14 (RpsN) | -6.22 |
| SP_0224 | 30S ribosomal protein S8 | -4.16 |
| SP_0225 | 50S ribosomal protein L6 | -4.88 |
| SP_0226 | 50S ribosomal protein L18 (RplR) | -2.64 |
| SP_0228 | 50S ribosomal protein L30 | -8.27 |
| SP_0229 | 50S ribosomal protein L15 | -4.51 |
| SP_0232 | Translation initiation factor IF-1 | -14.61 |
| SP_0234 | 30S ribosomal protein S13 (RpsM ) | -2.09 |
| SP_0264 | Proline-tRNA ligase | -3.10 |
| SP_0266 | Glutamine--fructose-6-phosphate aminotransferase | -2.32 |
| SP_0271 | 30S ribosomal protein S12 | -4.30 |
| SP_0294 | 50S ribosomal protein L13 (RplM) | -3.07 |
| SP_0350 | Capsular polysaccharide biosynthesis protein Cps4E | -3.73 |
| SP_0355 | Putative uncharacterized protein | -4.46 |
| SP_0373 | Putative uncharacterized protein | -2.30 |
| SP_0413 | Aspartokinase | -2.21 |
| SP_0425 | Acetyl-CoA carboxylase, biotin carboxylase (AccC) | -2.61 |
| SP_0433 | N utilization substance protein B homolog (NusB) | -2.43 |
| SP_0439 | Peptide chain release factor 3 | -4.06 |
| SP_0441 | 50S ribosomal protein L28 | -3.80 |
| SP_0443 | Putative uncharacterized protein | -2.34 |
| SP_0451 | Putative uncharacterized protein | -2.92 |
| SP_0493 | Probable DNA-directed RNA polymerase δ (RpoE) | -5.46 |
| SP_0494 | CTP synthase (PyrG) | -2.76 |

| Supplementary Table 2 continued | | |
|---|---|---|
| **SP #** | **Protein** | **Fold change** |
| SP_0564 | Putative uncharacterized protein | -2.72 |
| SP_0613 | Metallo-beta-lactamase superfamily protein | -2.17 |
| SP_0630 | 50S ribosomal protein L11 | -11.89 |
| SP_0631 | 50S ribosomal protein L1 | -7.14 |
| SP_0648 | Beta-galactosidase (BgaA) | -2.45 |
| SP_0670 | Putative uncharacterized protein | -4.17 |
| SP_0675 | Oxidoreductase, short chain dehydrogenase/reductase family | -2.80 |
| SP_0678 | Putative uncharacterized | -2.86 |
| SP_0702 | Orotate phosphoribosyltransferase (PyrE) | -2.57 |
| SP_0752 | Branched-chain amino acid ABC transporter, ATP-binding protein (LivG) | -2.65 |
| SP_0756 | Cell division ABC transporter, ATP-binding protein FtsE | -8.59 |
| SP_0761 | ATP-dependent RNA helicase, DEAD/DEAH box family | -2.87 |
| SP_0762 | S-adenosylmethionine (MetK) | -2.76 |
| SP_0770 | ABC transporter, ATP-binding protein | -3.08 |
| SP_0780 | Ribonucleoside-diphosphate reductase, alpha subunit, truncation | -2.83 |
| SP_0788 | Methionine-tRNA ligase | -3.87 |
| SP_0801 | Putative uncharacterized protein | -5.11 |
| SP_0838 | 30S ribosomal protein S20 | -13.86 |
| SP_0846 | Sugar ABC transporter, ATP-binding protein | -2.47 |
| SP_0852 | DNA topoisomerase 4 subunit B (ParE) | -2.36 |
| SP_0855 | DNA topoisomerase 4 subunit A (ParC) | -2.70 |
| SP_0861 | Putative uncharacterized protein | -2.62 |

| Supplementary Table 2 continued | | |
|---|---|---|
| SP # | Protein | Fold change |
| SP_0868 | Putative uncharacterized protein | -2.65 |
| SP_0871 | Putative uncharacterized | -3.67 |
| SP_0875 | Lactose phosphotransferase system repressor | -5.18 |
| SP_0876 | Tagatose-6-phosphate kinase | -2.88 |
| SP_0877 | PTS system, fructose specific II ABC components | -3.59 |
| SP_0897 | Pyruvate kinase (Pyk) | -2.18 |
| SP_0908 | Putative transcriptional regulator | -3.82 |
| SP_0923 | Cof family protein | -2.04 |
| SP_0929 | Pseudouridine synthase (RluD) | -2.10 |
| SP_0959 | Translation initiation factor IF-3 | -3.87 |
| SP_0964 | Dihydroorotate dehydrogenase B (NAD(+)), (PyrDB) | -6.16 |
| SP_0969 | GTPase Era | -2.36 |
| SP_0985 | Cys-tRNA(Pro)/Cys-tRNA(Cys) deacylase | -4.53 |
| SP_1010 | Large conductance mechanosensitive channel protein MscL | -2.35 |
| SP_1025 | Putative uncharacterized | -2.73 |
| SP_1068 | Phosphoenolpyruvate carboxylase (Ppc) | -2.83 |
| SP_1087 | ATP-dependent DNA helicase PcrA | -3.02 |
| SP_1093 | Putative uncharacterized protein | -5.41 |
| SP_1095 | Ribose-phosphate pyrophosphokinase 2 (Prs2) | -2.41 |
| SP_1107 | 50S ribosomal protein L27 | -4.56 |
| SP_1128 | Enolase | -2.75 |
| SP_1155 | Ribosome biogenesis GTPase A | -2.01 |
| SP_1182 | Lactose phosphotransferase system repressor (LacR) | -2.58 |
| SP_1243 | Glucose-6-phosphate 1-dehydrogenase (Zwf) | -2.14 |

| Supplementary Table 2 continued | | |
|---|---|---|
| SP # | Protein | Fold change |
| SP_1249 | GMP reductase (GuaC) | -2.56 |
| SP_1267 | LicC protein | -2.36 |
| SP_1269 | Choline kinase (Pck) | -3.63 |
| SP_1270 | Alcohol dehydrogenase, zinc-containing | -5.43 |
| SP_1275 | Carbamoyl-phosphate synthase large chain | -4.89 |
| SP_1276 | Carbamoyl-phosphate synthase small chain | -4.96 |
| SP_1278 | Bifunctional protein PyrR | -2.61 |
| SP_1293 | 50S ribosomal protein L19 (RplS) | -3.05 |
| SP_1297 | Flavodoxin (Fld) | -2.25 |
| SP_1362 | Adapter protein MecA (MecA) | -2.04 |
| SP_1397 | Phosphate import ATP-binding protein PstB 2 | -2.51 |
| SP_1421 | Nicotinate phosphoribosyltransferase | -2.83 |
| SP_1475 | Glycine--tRNA ligase alpha subunit (GlyQ) | -2.19 |
| SP_1483 | ATP-dependent RNA helicase, DEAD/DEAH box family | -5.25 |
| SP_1510 | ATP synthase subunit alpha (AtpA) | -2.43 |
| SP_1511 | ATP synthase subunit delta (AtpH) | -2.14 |
| SP_1521 | UDP-N-acetylmuramate--L-alanine ligase (MurC) | -2.28 |
| SP_1530 | UDP-N-acetylmuramoyl-L-alanyl-D-glutamate--L-lysine ligase | -4.26 |
| SP_1538 | Cof family protein/peptidyl-prolyl cis-trans, cyclophilin | -2.92 |
| SP_1539 | 30S ribosomal protein S18 (rpsR) | -2.31 |
| SP_1542 | Asparagine-tRNA ligase | -3.78 |
| SP_1557 | DegV domain-containing protein | -2.65 |
| SP_1573 | Lysozyme (LytC) | -7.32 |
| SP_1580 | Sugar ABC transporter, ATP-binding protein (MsmK) | -2.17 |

| Supplementary Table 2 continued | | |
|---|---|---|
| SP # | Protein | Fold change |
| SP_1584 | GTP-sensing transcriptional pleiotropic repressor CodY | -4.23 |
| SP_1586 | Probable ATP-dependent RNA helicase (Exp9) | -2.36 |
| SP_1631 | Threonine-tRNA ligase (ThrS) | -2.02 |
| SP_1667 | Cell division protein FtsA | -3.04 |
| SP_1674 | Putative phosphosugar-binding transcriptional regulator | -2.54 |
| SP_1701 | Phospho-2-dehydro-3-deoxyheptonate aldolase | -2.54 |
| SP_1702 | Protein translocase subunit SecA 1 | -3.05 |
| SP_1737 | DNA-directed RNA polymerase subunit omega (RpoZ) | -3.00 |
| SP_1739 | Ribonuclease Y | -4.23 |
| SP_1749 | GTP-binding protein | -2.40 |
| SP_1837 | Putative capsular polysaccharide biosynthesis protein | -2.96 |
| SP_1853 | Galactokinase | -10.13 |
| SP_1880 | Non-canonical purine NTP pyrophosphatase | -2.73 |
| SP_1882 | UPF0154 protein | -2.54 |
| SP_1888 | Oligopeptide transport ATP-binding protein (AmiE) | -2.18 |
| SP_1894 | Sucrose phosphorylase (GtfA) | -10.14 |
| SP_1923 | Pneumolysin (Ply) | -2.18 |
| SP_1937 | Autolysin | -10.98 |
| SP_1940 | Protein RecA | -2.36 |
| SP_1960 | DNA-directed RNA polymerase subunit β | -4.91 |
| SP_1961 | DNA-directed RNA polymerase subunit beta (RpoB) | -3.03 |
| SP_1970 | Aspartate-ammonia ligase (AsnA) | -2.50 |
| SP_1975 | Membrane protein insertase (YidC1) | -2.24 |
| SP_1978 | Diaminopimelate decarboxylase (LysA) | -3.04 |
| SP_1979 | Pur operon repressor | -7.99 |

| Supplementary Table 2 continued | | |
|---|---|---|
| **SP #** | **Protein** | **Fold change** |
| SP_1999 | Catabolite control protein A (CcpA) | -2.62 |
| SP_2030 | Probable transketolase (Tkt) | -2.01 |
| SP_2058 | Queuine tRNA-ribosyltransferase (Tgt) | -2.80 |
| SP_2076 | DNA mismatch repair protein HexA | -2.31 |
| SP_2078 | Arginine-tRNA ligase (ArgS) | -2.11 |
| SP_2121 | Histidine--tRNA ligase | -4.39 |
| SP_2135 | 50S ribosomal protein L33 type 3 (RpmG3) | -3.08 |
| SP_2156 | SPFH domain/Band 7 family | -2.81 |
| SP_2190 | Choline binding protein A (PspC) | -3.10 |
| SP_2204 | 50S ribosomal protein L9 | -4.24 |
| SP_2230 | ABC transporter, ATP-binding protein | -3.52 |

Only proteins with a decreased fold change of 2 and above are shown
